# Debilitated Lifeworlds: Women's Narratives of Forced Sterilization as Delinking from Reproductive Rights

**DOI:** 10.1111/maq.12700

**Published:** 2022-03-10

**Authors:** Julieta Chaparro‐Buitrago

**Affiliations:** ^1^ The Department of Sociology University of Cambridge

**Keywords:** debilitated lifeworlds, forced sterilization, reproductive rights, delinking, decolonial reproductive studies

## Abstract

Peasant women in Cajamarca, Peru, who were sterilized by the Peruvian government in the 1990s, narrate their experiences of reproductive abuse using Andean medical principles of *debilidad* and *fuerza* (debility and strength) (Tapias 2006). In their narratives, many describe a generalized sense of loss of strength resulting from the procedure. This contrasts with the reproductive rights framework's emphasis on infertility as the main harm. In this article, I ponder the dissonance between these two frameworks and propose the concept of debilitated lifeworlds as decolonial feminist delinking (Mignolo 2007) from human fertility‐centric narratives. This concept is methodologically significant as a decolonial attunement to local motifs to talk about abuse and for weaving a constellation of embodied, emotional, social, and family harms. This article contributes to the emerging field of “decolonial reproductive studies” (Smietana et al. 2018: 117).

## Introduction

Under the blue skies and a chilled breeze characteristic of the winter season in the Andes, Luz and I sat down to talk about her story of being sterilized back in the 1990s, when former president Alberto Fujimori launched an aggressive family planning program to reduce poverty indicators in Peru. Luz arrived at my doorstep after she heard me on the radio the night before. During the evening show, I explained the purpose of my visit to Cajamarca: I wanted to talk to women who had been sterilized two decades ago. She showed up the next morning because she wanted to be interviewed. I rushed back to my room and brought out my voice recorder and notebook, and we sat at one of the corners of the courtyard where we found refuge from the blazing sun. She took me with her into her memories. She remembered going to the health center to get a refill for contraceptive pills. Instead, Leticia, one of the nurses, told her it was better to get a *ligadura*.^1^ Luz repeated her words: “You will not survive another pregnancy, and you will not have money to support all your children.” Luz pondered the fate of her little ones without a mother. “They are going to suffer without me,” she thought. The nurses told her to come back to the health center for the surgery.

A couple of days later, Luz showed up feeling happy about receiving medical care and motivated by Leticia's promise of weight gain and strength. As Luz waited for her turn sitting next to other women, the medical personnel carried out a woman from the operating room “whose head was hanging to the side as if she was dead. I got scared, and I wanted to leave. I begged the nurses to let me go because I no longer wanted to get the ligadura, but they didn't listen to me. Open the door!, I said many times, but they didn't do it.” Instead, Leticia insisted on the sterilization procedure, arguing she would not live through another pregnancy. Quickly, they took her to the operating room, violently tied her hands and feet to the bed, and prepared her for the surgery. She woke up with two bandages on her belly.

After the operation, Luz started having back pain that left her unable to work for two years. The sterilization marked a turning point in her life that brought a sense of general weakness she described as *invalidez* (invalid) that prevented her from carrying out daily tasks at home and in her chacra.^2^ Part of the money her husband made was used to pay for medicine to ease the pain, and this angered him, causing tension between them. “He called me a ‘bad woman’ and many times I thought of leaving. My head was spinning with lots of *preocupaciones* (worries). I resembled a drunken person. I used to knit and braid hats, but not anymore. It hurts a lot.”

Luz's story is similar to the stories of other women I interviewed and spoke with who experienced a sense of loss of strength after they were sterilized. They refered to a generalized sense of physical weakness that limited their capacity to do physical labor. Peasant women narrated their experience of reproductive abuse through Andean medical principles of *debilidad* and *fuerza* (debility and strength) (Tapias 2006) that shaped how they underwent the procedure and made sense of it in their narratives (Kleinman 1988). Under the human and reproductive rights framework, however, loss of strength is taken for granted and displaced by an emphasis on infertility as the main harm.

The ability to control one's fertility is one of the cornerstones of reproductive rights as defined in the Platform of Action of the International Conference on Population Development (United Nations Population Fund 2014). It refers to the right of couples and individuals

to have a satisfying safe sex life and … the capability to reproduce and the freedom to decide if, when, and how often to do so. Implicit in this last condition are the right to … be informed and to have access to safe, effective, and affordable methods of family planning of their choice … for regulation of fertility which are not against the law. (United Nations Population Fund 2014: 59)

Fertility becomes a legally protected good; thus, it is safe to suggest that acts that coercively end one's fertility constitute a reproductive rights violation.

The dominance of infertility has shaped public discourse about forced sterilization in Peru. Two examples instantiate this. In the opening lines of photographer Tadeo Burbon's (2018) column *Entre el Despojo y la Espera*, he writes, “those responsible for the Peruvian case of forced sterilization deprived thousands of women and men of their reproductive capacity, stripping them off of this biological property with impunity” (Burbon 2018, my translation). Leaving women unable to reproduce is, according to Bourbon, the foremost violation. In his photography series *Pecati, Esterilzaciones Forzadas en el Perú*, included in the exhibition *Ikumi* at the Place of Memory, Tolerance, and Social Inclusion in Lima, Burbon tethers sterilization to fertility, arguing that it “deprived women of their ability to be mothers” (Burbon 2020).

*Cicatrices del Engaño* (Scars of Deception) (Hiperactiva Comunicaciones 2014) was part of a wave of documentaries, including Una Voz Esteril (Ruiz 2014) and *La Cicatriz de Paulina* (2010), produced in the early 2010s, documenting the testimonies of survivors of forced sterilization. A scene in *Cicatrices* juxtaposes the statement of a survivor, Concepción Bellido, who describes the impact of the ligadura on her ability to work, to the explanation of an NGO worker that emphasizes infertility. As Concepción explains:

I feel bad, I cannot lift heavy things, I cannot work on the chacra. Before, I could do it normally. I feel bad, I feel. … I get chills. Why did they have to marginalize us [sobbing] and send those programs that harm us [sobbing and rubbing her eyes]? We are marginalized, criminalized (*sic*). Let it be justice for us, miss (Hiperactiva Comunicaciones 2014, 4:50 to 5:19, my translation)

After Concepción's testimony, the documentary introduces the statement by an NGO worker who explains the significance of the procedure by privileging the rhetorical grid of infertility. “In the Andean culture,” the NGO worker notes,

the linkage between a woman's womb with the earth, with the fertile soil is strong. The worldview is also built upon this principle, the womb as the Pachamama [mother earth]. So, by taking away from them the possibility of having children, the possibility of deciding about their bodies and mothering is brutal what happens to all of them (Hiperactiva Comunicaciones 2014, 5:55 to 6:32, my translation).

While Concepción's explanation highlights the loss of strength, the NGO worker's explanation displaces it and recenters fertility as a legible framework for understanding sterilization abuse.

Although women's testimonies include various sequelae^3^ besides infertility, these are often left unattended under the framework of reproductive rights and fact‐finding activities. As legal scholar Fionnuala Ní Aoláin (2016) notes, fact‐finding selectively documents harms women have had to negotiate in their lives. A postcolonial critique, then, questions which harms get documented to position violence against women in political and legal agendas. A closer look at the gendered politics of forced sterilization fact‐finding reveals the heteronormativity and repronormativity (Franke 2001) underlying women's social identities. Infertility is viewed as analogous to disability or chronic illness, and is a source of social stigma for those who are unable to fulfill motherhood as an expected role (Greil 1991). The gendered politics of forced sterilization underlies the emphasis on infertility as the main harm.

In this article, I go beyond a postcolonial critique to propose a decolonial reproductive framework. The issue at stake is not merely the reductionist character of fact‐telling narratives, but the hierarchy of value (Ní Aoláin 2016) between overlapping narratives of forced sterilization. Women's narratives of debility and reproductive rights stand in a stratified relationship to one another, creating a dissonance between the dominant explanatory framework of harm and the lived experience. In this article, I propose the concept *debilitated lifeworlds* as a decolonial feminist delinking (Mignolo 2007) from fertility‐centric narratives of reproductive rights. It is a gestural displacement (Schechter 2017) that does not take infertility as the singular point of departure for understanding the multiple harms resulting from sterilization abuse. Thinking with debilitated lifeworlds allows me to map out a dense network of experiences that connect the loss of fertility, loss of strength, and social and emotional turmoil.

This article is based on ethnographic fieldwork conducted between 2016 and 2017, including 18 semi‐structured interviews and informal conversations with survivors in the northern department of Cajamarca. We discussed the sterilization procedure and their experiences before and after. The women I spoke with were in their 40s to early 60s. Their daily lives revolved around agricultural activities, tending livestock, and raising small animals. In addition, some of them participated in the Saturday market, either selling animals, produce, or prepared foods. Two of them ran small businesses, including a restaurant and an arcade. Some women were also involved in local politics as *regidoras* (local councilors) and members of community committees. I complement the ethnographic material with archival documentation by the Ministry of Health, including policy documents and implementation handbooks, as well as visual material available online. In my analysis, I explore the relationship between tubal ligation and a sense of weakness and generalized debility, its connection to other physical and emotional harms, and how to account for them, mapping out a fuller landscape of reproductive violence.

Debilitated lifeworlds resonate with other postcolonial, decolonial, and queer feminist analyses that critically assess the exhaustion and debilitation of racialized bodies as part of neoliberal biopolitics and labor regimes (Livingston 2005; Puar 2017; Vergès 2020). Historian Julie Livingston (2005) traces the socio–political and economic transformations in Botswana after decolonization through people's experience of debility, including the increase and dominance of the migrant labor system, religious conversion to Christianity, and transformation of the health care system. Debility, Livingston explains, is not simply about physical impairment; it is also about history, social relations, and bodies. Revisiting biopolitics not simply as a project “to make live” but also as one to “not let die,” Jasbir Puar (2017: x) argues that racialized bodies are injured and debilitated as a means of exercising control over them.

Debilitation, Puar (2017) notes, is not equivalent to disability as an event. Instead, it names the long‐lasting exposure to violence that wears down bodies and makes them weak. Debilitation is not an identity but the result of economic and political calculations on certain bodies that “are expected to endure pain, suffering, and injury” (Puar 2017: xiv). According to Françoise Vergès (2020), the racialized bodies of women who make up most of the ranks of the working class, work late at night or early in the morning to prepare the spaces for the functioning of patriarchal neoliberal capitalism. Their bodies are exposed to toxic products and exhaustion, and they receive meager paychecks for work that is considered unskilled, yet fundamental to sustaining the efficient bodies of the neoliberal bourgeoisie. My work expands on these insights to show how lifeworlds, not only bodies, are subjected to long‐lasting debilitation through forced sterilization.

## The Reproductive Health and Family Planning Program, 1996–2000

Former Peruvian president Alberto Fujimori announced the implementation of the Reproductive Health and Family Planning Program (RHFPP) during his presidential speech in 1995 after he was reelected by a landslide. Despite the self‐coup in 1992 that closed down Congress and gross human rights violations during this first term, Fujimori enjoyed smashing popularity. He claimed he had pacified Peru after more than a decade of armed conflict and alleviated some of the effects of what had been, since the mid‐1980s, a dire economic situation. These were volatile years in Peru. With armed confrontations between the army and rebel forces, inflation rates that skyrocketed, and almost 50% of Peruvians living in poverty, Fujimori found in population control an “easy fix” to such a complex scenario. Ironically, this happened amid the consolidation at the UN of the reproductive rights and health framework that discredited aggressive population control programs and made visible the abuses committed in places like India and Bangladesh in the previous decades.

The novelty of this program was its incorporation of the notion of women's empowerment into the government's agenda of poverty reduction (Chaparro‐Buitrago 2019). The state would be the guarantor of empowerment by allowing women to control their bodies and reproductive lives as a means for lifting themselves out of poverty. Unfortunately, the program turned out to be a platform for unconsented sterilization, targeting mainly indigenous, peasant, and low‐income women across the country. According to the Peruvian Office of the Ombudsman^4^ (Defensoría del Pueblo 2002), more than 200,000 low‐income and indigenous women were sterilized in four years, many of whom were bribed, deceived by health care providers, and, in some cases, forcibly sterilized in health centers.

Feminist lawyer Giulia Tamayo, one of the first to investigate the abuses, suggests in her report *Nada Personal* (CLADEM 1999) that during the implementation of the RHFPP, the cultural worlds and meanings peasant and indigenous communities attribute to the body and reproduction were overridden by the imperative to reduce fertility rates. Although loss of strength is often dismissed as a folk belief or even a lie, it was recognized by the Ministry of Health as a potential deterrent to operation. The *Manual de Normas y Procedimientos para Actividades de AQV* (Ministerio de Salud 1996) lists “myths and rumors” associated with tubal ligations and vasectomies, and it instructs providers on how to “correct” them during counseling sessions. Some of the “erroneous concepts” associated with female sterilization include the idea that the procedure is painful and difficult; that it makes women weak, alters their character, makes them crazy, or causes loss of sexual drive; as well as the idea that the fallopian tubes can be untied after seven years. Among the myths associated with vasectomies are the fear of castration and loss of sexual appetite, weight gain, loss of muscular strength, and weakening of the immune system. Contrary to the characterization of these ideas as erroneous concepts that need to be rectified, in the next section, I show that they reveal a horizon of meaning through which women experience and make sense of the procedure.

## “Ya no Tenemos Fuerza”

Amalia and I arrived at Gloria's doorstep. She invited us in as her husband is gone, freeing some time for chatting. She brought two pieces of sheepskin and two small stools for us to sit on. Her eyes smiled. She was pleasant, and her voice came out softly, with a shade of sorrow in it. Gloria recalled multiple visits from nurses to her house back in the 1990s to talk to her about the sterilization. They suggested she already had too many children and mentioned an alleged tax for families with more than four infants. Gloria was afraid of the operation mostly because she thought her husband would turn jealous and violent because of fears of infidelity after the procedure and because she did not fully understand it. The nurses insisted that the sterilization was a safe procedure and assured her she would be back home the next day to tend to her family. “You will be up and cooking the next morning. No need to be afraid,” they reassured. Despite her fears, Gloria accepted and went to the health center to get the procedure done. She was in bed for 11 days after the surgery with a burning sensation in her belly. For years, she could not walk long distances or sit up straight, had constant headaches and *preocupaciones*, and persistent worries about her health and the fate of her children, which left her unable to sleep at night. As a result of the operation, Gloria said she lost strength, and until today, she limits how much physical effort (fuerza) she makes.

Gloria's story elicits a key question: What is the relationship between a cut in the belly area and the ensuing sense of debility and weakness? In a broad sense, a cut in the belly area has debilitating effects because it is the site of the body where productive and reproductive capacities reside. The uterus, also referred to as *madre* (Sp. mother, Qu. madri. Womb. Larme 1993), is the place of vital force and bodily vigor. Women experienced the cut of the abdominal area as a disturbance of their physical strength, mainly because it disrupted life's vital activities such as work, sex, and reproduction, all of them associated with basic processes for sustaining life.

Biocultural and medical anthropologists have documented reproductive‐related illnesses linked to distress in the Andes and Mesoamerica. *Teta asustada* (Theidon 2013), *susto, madre onqoy, sobreparto* (Kuberska 2016; Larme 1993; Larme and Leatherman 2003; *Yon* *2000*), *sopla* (Larme 1993), *debilidad* (debility, weakness) (Oths 1999; Yon 2000), *isihuayo* (Smith‐Oka 2014), and *prolapso* (Yon 2000) feature among the most common ones. They represent cultural models of health in which biological, psychological, somatic, and socioeconomic, political, and supernatural elements are intertwined in multi‐causal explanations (Larme 1993; Oths 1999). Anthropologist Anne Larme (1993) suggests that reproductive illnesses are deeply intertwined with women's work, as reproductive processes impact women's bodies and their capacities to perform (re)productive tasks. I understand the loss of strength both as a social and embodied experience and as an “idiom of distress” (Lock 1993: 142) “available to communicate bodily states, including states of illness” (Kleinman 1988: 13).

Rural communities in Mexico and Peru link together (re)production, body vigor, and the uterus (Larme 1993; Oths 1999; Smith‐Oka 2014; Yon 2000). In her analysis of isihuayo among Nahua women in Mexico, Vania Smith‐Oka (2014) describes it as an illness of organ displacement that causes health problems. Isihuayo causes the uterus to drop down due to overexertion during labor and everyday work. Nahua women believe that carrying heavy loads weakens their uterus and bodily vigor over time, especially during pregnancy and early post‐partum (Smith‐Oka 2014). Debilidad is an ailment that affects women (and men in fewer numbers) in northern Peruvian highlands who have completed their reproductive roles (Oths 1999). Like isihuayo, debilidad results from the physical and psychological effects of intense physical labor, repeated childbirth, and loss (Oths 1999). Peruvian anthropologist Carmen Yon describes similar findings among Quechua and Aymara women in southern Peru. Having multiple births deteriorates women's health as they “mistreat the body and make the womb or the ovaries swell.” These are considered signs of future reproductive illnesses such as *regla blanca* or *cáncer de madre* (Yon 2000). Larme (1993) finds among women in the southern department of Puno (Peru) the idea that the madre is perceived as a delicate part of women's bodies, and that further trauma debilitates it.

The women I interviewed provided a body map of the symptoms they experienced as a result of the sterilization: headaches (often at the back of the head as women indicated by touching it, and sometimes more specifically described as brain pain), along with body pain (lower abdomen and lower back, hands, and feet). Some also described feeling *como borracha* (like drunk), and unable to fall asleep at night. This collection of bodily sensations resemble debilidad's symptom profile, which includes headaches, mostly brain pain and headache in the cranium, as well as bodily complaints such as aches, chills, and numbness, dimmed eyesight, dizziness, sleep disorders, and loss of appetite (Oths 1999).

According to Oths (1999), women and men at (re)productive age (women between 20 and 31 and men between 21 and 45) tend not to experience debilidad until somewhat later in life. This contrasts with the profile of women who were sterilized during the 1990s. At the time of the interviews, women were between their 40s to early 60s, indicating that they were sterilized when they were expected to bear children and care for them, and to contribute to the family economy. If debilidad is prevalent among women who have finished their reproductive lives, the distress brought by the premature interruption of women's reproductive cycle is expressed through symptoms that mirrors it.

The difference, however, is that loss of strength is not about a body that has been worn down by cumulative hardships, as in debilidad or madre. It is instead about a body that has been *malogrado*, *baldado* (damaged caused by an external cause) and left *inservible* (useless), contrary to health care providers’ promises that women would be back on their feet next day “as if nothing had happened,” feeling healthy and vigorous. Gloria experienced excruciating pain after the surgery, a burning sensation in her belly, and headaches. These symptoms unleashed constant worry about her health and the fate of her children. Even though giving birth has deteriorating effects on women's strength and vigor because it weakens the madre (Larme 1993), Gloria would have preferred consecutive birthing. It wouldn't have had immediate adverse effects on her strength. “A thousand times,” she said, “I would have given birth to lots of children. Eight days after we give birth, we are walking and doing our things. But this is worse. It burns, hurts, bad.”

Many women recounted with sadness and shame being called *haraganas* (sluggard) by members of their communities for not being able to work, which was (and still is) considered a discrediting personality trait. More importantly, not being able to do the heavy lifting in their chacras or walking for long hours made the job of sustaining their families more onerous. Families in Cajamarca follow a vertical model of production, living in a home community and traveling to subsistence zones located at different altitudes where they harvest their produce (Larme 1993). Women had to walk long distances to harvest ocas and potatoes in high‐altitude areas like la Jalca, located a 4.5‐ to 5‐hour walk uphill from their home. This task became more demanding after the procedure. Additionally, most husbands were away for periods of four to six months, as they left to work as seasonal laborers in coastal plantations, in mines, or as traders. Women stayed at home and were responsible for feeding the children and taking care of agricultural tasks alone. Although many of them had been responsible for a significant amount of these chores, their partner's absence felt even stronger after they experience loss of strength.

As we have seen, the issue at stake is not merely that women lost their reproductive capacities, but that they also lost the strength to perform productive labor. As anthropologist Gayle Rubin (2014) argues in her classic essay “The Traffic in Women: Notes on the Political Economy of Sex,” the distinction between economic and sexual systems that links the economy to production and sexual systems to reproduction is flawed. It is problematic to relegate all aspects of reproduction to the sex system and limit sex to biological reproduction. One attempt at bringing production and reproduction together has been to consider reproductive activities (pregnancy, childbirth, and breastfeeding) as forms of labor. These are demanding tasks for women performed alongside economic activities that “affect women's abilities to carry out other tasks … and [their] overall health status” (Larme 1993, 86). In her ethnography of market women in Huaraz (Peru's Central Andes), anthropologist Florence Babb (1998) pushes the argument further. She breaks down the binary of productive and reproductive labor, showing that women's work plays an essential role in reproducing household labor and in production within the social and economic sphere. Because of the mutual interdependence of productive and reproductive labor, the cut in the belly area represents a disruption in both women's fertility and their capacity to work.

Women identified the operation as the cause of lost strength, often contrasting their well‐being before and after the procedure. This temporal element was key to understanding the experience of loss of strength that resulted from the disruption of fertility, vitality, mobility, and work. Women's narratives conveyed a nostalgia for a past where they were *recias*, a term women use to describe a healthy, robust, and vigorous person capable of performing all the chores associated with peasant life: cook, fetch wood, clean, wash clothes, herd and take care of animals, cultivate the land, walk long distances, along with raise children. Luz put it as follows “I was able to lift heavy weight, I used to harvest my corn and my potatoes by myself. I also carried my baby on my back. I took my horse with me and loaded him with all the produce, with a big sack of potatoes. After the operation, I was left *invalida* (disabled). Para hacer fuerza ya no estoy, señorita.” The word invalida indicates Luz's perception of the sterilization as an injury that made her weak and left her unable to move and carry on with the daily activities. Sadness and sorrow shaped women's nostalgic accounts, exacerbated by a sense that the debility brought by the sterilization was compounded with an already precarious economic situation. Later in the interview, Luz speculated about what her current life would be if she hadn't had the operation. As she poignantly puts it:

I would be recia, I would be able to go to the coast and find a job, as many women my age do. I would be working in the fields, collecting tomatoes, chilies, maybe corn or onions. Sometimes I go there with my daughters who work in the plantations. I take care of my grandchildren while they work and when I look at them from afar I cannot help but to regret [the operation].

The long‐lasting effects of loss of strength arose from the idea that the wounded uterus never fully heals and it could open at any moment, mostly when they performed tasks that require physical strength. Gloria explained that when she “made physical effort” (fuerza) she felt her belly blistered inside, and she was afraid it would burst. Her belly burnt (*ardía*) when she did heavy work, and, to avoid the discomfort, she wrapped her belly to prevent the wound from reopening.

When I bind myself, I can work with my husband in the fields. If I have a cow, I milk it, and then we go to check on their other animals. I help my husband chop off wood. Sometimes we have large pieces, and we use the machine to break them down. Today he went up there [pointing to a mountain] to bring more wood. Tonight, we'll cut it to make furniture for sale.

The idea of the body being open due to the operation resonates with Andean notions of body openings, such as the head, orifices like the ears, anus, and vagina, the lower back, and feet that render the body vulnerable to illnesses (Larme 1993). The cut of the tubal ligation creates an extra opening in an area of the body that is already considered fragile. Gloria binded her abdomen as the cut never fully heals, leaving her uterus feeling tender and weak. A girdle prevented the wound from reopening and helped her to continue with her routine.

## Interconnected Harm(s): Loss of Strength, Conflicts at Home, and Preocupaciones

It would be a mistake to suggest that loss of strength is isolated from other social and emotional harms of the sterilization. Following legal scholar Fionnuala Ni Aoláin's (2016) call for exploring the interconnection between various forms of harm that correspond to structural conditions of inequality, in this section, I map out the social and emotional harms connected to loss of strength. For almost all the women I spoke with, sterilization left a mark on their family and community life. It unleashed conflicts at home, especially with partners and other family members who perceived them as a *carga*, a burden to the family. Their children, now young adults, also question their mothers for having accepted the procedure.

Gloria, Virginia, and Luz explained that their husbands disowned them for not being able to work. Virginia's husband used to tell her, “You are worthless. You used to be recia like other women who do not whine about pain. Live as you can. There is no money.” Virginia recalled his bitterness and the constant fighting at home. She tried to explain that she felt lonely, but he kept condemning her for not having more children to work with him in the chacra. In Gloria's case, her husband's family urged him to leave her because of the money he had to spend on her recovery. She faced episodes of domestic violence when the initial fear about her husband's reaction turned into black eyes and an escape from home in the middle of the night seeking refuge at her sister's house.

Paulina also refered to conflicts with her son and daughter, who reproached her for getting the procedure, revealing a lack of understanding of the coercive context in which it took place. They yearned for another sibling they could count on and confide their problems to. Paulina also felt lonely at home after her children moved out, and she spent most of her days alone taking care of her animals. Although she still lives with her husband, he is away during the weekdays because of his job as a trader. For Paulina, the ideal family size is four children; five, she said, is too much. Had she had two more children, she would still have at least one to keep her company.

The physical harms have an emotional correlate. *Preocupaciones* (worries), also referred to as *pensamientos* (thoughts), are the emotional wounds of abusive sterilizations. These “emotional thoughts,” as medical anthropologist Kimberly Theidon (2013: 41) describes them, “blur the distinction between intellectual and affective faculties.” Preocupaciones were described by the women I interviewed as a state of restlessness and nervousness caused by possible problems the sterilization could cause. Luz described them as an internal conversation, thoughts about death and abandonment of her children that rushed through her head. Other women mentioned that preocupaciones came especially at night when they went to bed, leaving them sleepless. Preocupaciones never entirely disappear. The intensity might lessen, but worries about poverty and financial insecurity persisted. As Luz explained, the cut eventually healed; however, “I am not fully healthy, I worry. I cannot forget what happened.” Preocupaciones had a bodily manifestation in symptoms such as constant headaches, foggy mind, not being able to “think straight,” uncontrollable crying, and sleeplessness. As Gloria described it, “I felt dizzy, as if I were drunk all the time.” All these symptoms had a debilitating effect and could be another cause behind the loss of strength: with little sleep, women did not have enough energy to take on daily tasks.

Not all women I spoke with experienced loss of strength, although those cases were a minority compared to the overwhelming number of women who critically assessed the ligadura as abusive, or as Luz lucidly put it, when the government “play[ed] with our bodies.” One of the two women I spoke with who did not feel débil was Emelia. We spoke in her home after Amalia introduced us at the Saturday market. We sat down under the shade of a porporo tree while eating fruits that her husband had pulled down for us. “Some women felt bad after the surgery, but I didn't feel anything,” Emelia told me. Not feeling the operation meant that Emelia did not experience weakness or debility. She got the ligadura after the health care director insisted she had too many children and urged her to get the procedure done. After the operation, Emelia's mother took care of her in her husband's absence, who went to the coast for work, helping with the house chores and childcare. After a month, Emelia remembered going to La Jalca on her own to harvest ocas and potatoes that she brought back to the house.

Emelia's story reveals two things. First, there is a correlation between the experience of loss of strength and debility and the perception that the sterilization was abusive—even though she was sterilized without a counseling or reflection period. Second, strength and debility are the frameworks through which the procedure is experienced and narrated. Emelia and Julia, the only two who did not experience loss of strength, also reported that they wanted to get the procedure to control their fertility. They had reached their desired fertility and were facing economic precarity and conflicts with partners, and they felt overburdened by their workload at home, including childcare.

Loss of strength is not merely a somatic expression of psychological disturbances, which reduces the explanation to individual psychological states. It is a set of symptoms with communicative effects that requires an analysis of the role that loss of strength plays in women's narratives. As medical anthropologists have long suggested, bodily symptoms respond to social, political, and economic factors, and local environments (Kleinman 1988; Leatherman 1998) that frame the experience of health and disease at the bodily level (Henry 2006; Jenkins 1991; Lock 1993). Together, these factors contribute to the reproduction of illness and poverty (Greenway 1998).

Medical anthropologists working in the Andes understand health as the outcome of larger socioeconomic structures, shaped by class, gender, and ethnicity (Leatherman 1998). Building on this idea, I suggest that narratives of loss of strength emerge from the encounter between Andean understandings of the body, social relations, and reproduction with larger structures of discrimination and inequality. Andean medical models configure the “contextual sociality of the body to discern how people internalize and embody the ‘externality’ of social relations” (Tapias 2006: 402). Debilidad and fuerza, as anthropologist Maria Tapias (2006) suggests, are used in the Andean world to describe a person's constitution or their histories of distress and illness. They constitute the grammar through which women communicate their experiences of reproductive violence, or to use Arthur Kleinman's words, debilidad y fuerza are “illness idioms … cultural orientations [that] organize our conventional common sense about how to understand and treat illness” (Kleinman 1988: 5). We can think of the loss of strength both as shaping women's embodied and social experience, as well as a locally available motif to address women's pain, discrimination, and vulnerability.

The introduction of an aggressive family planning program to solve the macroeconomic disaster after a decade of armed conflict, economic mismanagement, and the introduction of neoliberal reforms has multiple impacts. The embodied conjunction of macro socioeconomic transformations in Peruvian society has detrimental impacts on the racialized gendered bodies of peasant and poor women, troubling their sense of self and their familial and communal relations (Livingston 2005). Jasbir Puar's (2017) insight is key here to show how debilitation is neither an event nor a medical diagnosis but the historical exposure to conditions that weakens bodies. It is almost “a normal consequence” (Puar 2017: xvi) that some bodies are expected to endure. The violence in this expectation, as Puar puts it, is what debility instead of disability names. But as I have shown, debility is not simply about bodies. It has a much broader impact on lifeworlds, communities, and relationships.

## Debilitated Lifeworlds: Delinking from Reproductive Rights

The ethnographic material I presented above tells us a different story of reproductive abuse that contributes to a growing field that Smietana et al. have described as “decolonial reproductive studies” (2018: 117). I propose the concept of debilitated lifeworlds as an epistemological displacement, a feminist decolonial move to delink (Mignolo 2007) from fertility‐centric narratives of reproductive rights. Delinking from reproductive rights foregrounds their totalizing effect in representing harm as a single issue, in this case infertility, limiting our understanding of its interconnections with different aspect of people's lives. Likewise, delinking underscores the dual process of commensuration and displacement involved in making a whole constellation of abuses fit into the bounds of reproductive rights. Debility, as one of those harms that cannot be accommodated, is displaced or treated as excess of narratives, which, paradoxically, is one of the most significant outcomes of the sterilization in women's lives, as I have described in this article.

The dominant discourse of reproductive rights and the imperatives of judicial proceedings configure a stratification of discourses that leave unattended important aspects of women's narratives. This epistemological hierarchy not only renders invisible how women give an account of their experience, but also reinforces the idea that what is at stake is a single issue, such as infertility. Instead, the concept of debilitated lifeworlds highlights the mutual interdependence of infertility and debility, showing how their impacts exceed individual bodies to radiate into families and communal relations, and cautioning against taking for granted preconceived subjective and bodily experiences resulting from reproductive abuse.

Centering loss of strength allows me to explore a constellation of interrelated harms that women experienced after the procedure that is hardly accounted for by the reproductive rights and health framework. This framework consolidated at the United Nations’ ICPD and Beijing conferences. It recognizes people's right to reproduce and the freedom to decide the number of children they have, thus framing fertility as a protected status. Although reproductive rights and health mark an important development in the recognition of women's human rights legislation, it is also a source of dissonance with peasant women's lived experience.

Delinking allows me to show how particular understandings of the body, reproduction, and abuse, and the isolation of fertility from other areas of life, such as work and community life are imposed as a universal framework for recognizing reproductive injustices. In human rights legislation, forced sterilization constitutes a violation of physical integrity. As legal scholar Christyne Neff (1990) explains, physical integrity “safeguards the physical parameters of a person” (p. 328) “against non‐consensual contact or intrusion with any part of the body—inside or out— … This protection creates an inviolable shield around the body through which nothing may enter nor be extracted without consent” (Neff 1990: 339–40). Its focus on individual bodily harm imposes a particular understanding of the body as an isolated entity, devoid of social relations. This explains why the focus of reproductive violence on the physical harm inflicted on individual bodies, epitomized in the cut of the fallopian tubes as the cause of infertility.

Debilitated lifeworlds holds together the idea of sterility both as the inability to bear children and the inability to produce plants and crops, underscoring the artificial division between areas of life pertaining to production and reproduction. This concept, however, goes beyond individual embodied experiences, to weave a constellation of emotional, social, and familiar harms that radiate from and have repercussions on women's experience. Debilitated lifeworlds is about work, family, communities, and emotions, as much as it refers to bodies and fertility. It is also an invitation to think about fertility in a more expansive way. Fertility as vitality, as fuerza, as movement that is extracted from bodies, families, and land. Fertility as vitality is a shared quality of life, not simply a capacity that can be truncated.

Debilitated lifeworlds constitutes an “epistemic shift and brings to the foreground other epistemologies, other principles of knowledge and understanding” (Mignolo 2007: 453). Through this concept I show how Andean medical principles of debilidad and fuerza configure a different grammar to understand reproductive abuse. Loss of strength conveys the abusive character of tubal ligations, and its unfolding impacts on women's lives: loss of their economic and reproductive roles within the family, emotional turmoil, marital conflict, and stigmatization in their communities. It also connects the economy of sterility with the somatic, social, and emotional levels. The sterilization campaign was the result of a discriminatory health care system and a government that subjected peasant and indigenous women's lifeworlds to the logic of economic rationality, bundled up with the eugenic legacy to calculate who deserves to be born and who does not. In this case, such calculation was not uniquely based on someone's alleged “fitness” for parenting, but also on the demands of economic development and the pressures of population dynamics (Murphy 2017).

Implied in debilitated lifeworlds is the fact that not all are equally vulnerable to debilitation. The intersections of race, gender, and class exposed women like Paulina, Luz, and Gloria to coercive sterilization. Debilitation makes visible the unequal distribution of vulnerabilities along gendered and classed racism. As the exhausted and debilitated bodies of neoliberalism, debilitated lifeworlds are fundamental to the reproduction of economic agendas. Certain women's reproductive lives needed to be curbed as a precondition for economic development. These were the lives of indigenous, peasant, and low‐income women that were, paradoxically, a threat to economic development and the only way to achieve it.

Debilitated lifeworlds may not fully capture the tapestry of harms that unfold from reproductive abuse that other peasant indigenous women experience. However, I argue that this concept has a methodological significance as a decolonial attunement to local motifs used to talk about abuse, the social scaffolding that provides its meaning, and the larger socioeconomic and political context in which it takes place. This concept is an analytic for understanding reproductive abuse from a decolonial perspective that requires, as I argue in this article, decentering (without overlooking) fertility to understand its broader implications. This move cuts through the epistemological bounds of reproductive rights and it works to “de‐naturalize concepts and conceptual fields that totalize a reality” (Mignolo 2007: 459). This is particularly important when researching reproductive abuse against indigenous and peasant women, who have been disproportionately targeted (Gurr 2015; Theobald 2019). It is imperative to account for the frameworks that shape their narratives and how their lifeworlds are compromised.

One of the implications of my argument is that peasant or indigenous women tend not to use legal categories to account for their experience. This is often viewed as an indication of a lack of awareness that a violation occurred. Such a view reinforces a colonial imagery of indigenous or peasant women as passive, ignorant, and always victimized. Instead, a decolonial attunement opens space to understanding reproductive abuse otherwise, at the margins of legal concepts that reveal other ontologies at stake, and another set of principles and categories through which abuse and violence are experienced and accounted for.

## Final Thoughts

Conscious of the possible victimizing effect of zooming into the experience of debility and the knot of emotional and social harms associated with it, I warn against its inadvertent reinforcement. The women I worked with are mothers and grandmothers; they work hard in their chacras, take their products to the market, take care of their animals, and participate in local politics and women's organizations. They love being mothers, but they also feel the burden and the difficulties of raising children. In hindsight, Paulina saw the benefits of having fewer children, as she and her husband were able to provide better living conditions for their existing son and daughter. However, as she clarifies to prevent the erasure of the abuse and the violence, “it was not the way it should have been done. Had they talked to us nicely, explained the ligadura properly, and treated us well, things would have been different.” The experience of being sterilized is just a slice of women's lives. Undoubtedly, remembering animates feelings of sadness, sorrow, and anger for many. It is painful to go back to those years. Yet, they are much more than being sterilized.

My analysis unravels—albeit in a potentially incomplete way—the grammar through which women explain their experience of being sterilized by the Peruvian government two decades ago. A decolonial move in academia is paradoxical, as it seeks to make conceptual displacements that may assimilate difference within its frameworks. Building on the work of Helen Verran, Marisol de la Cadena and Mario Blaser (2018) suggest that epistemic explanations may translate difference back to its own terms, running the risk of canceling divergences and incommensurabilities. Debilitated lifeworlds is a gestural displacement to understand the experience of infertility and debility that intend to explain an embodied experience that seems to all others, except the women, almost incomprehensible. Incommensurability is at stake here, along with the possibilities of misunderstanding and misrepresentation, which are not exceptions but constitutive of ethnography work, yet rarely acknowledged (see De la Cadena and Blaser 2018).

The importance of this point becomes clear when examining the legibility of loss of strength outside of women's communities. I see the risks of its dismissal within human rights circles, where debility is turned into excess and often elicits pity as the emotional response. Within feminist circles, it is displaced by the rhetoric of reproductive rights and its almost exclusive focus on the state's role in robbing women of their fertility. Health care professionals often disregard the loss of strength as a folk belief, and suggest that women are just querulous and whiny. All these reasons explain the urgency of a decolonial take on reproductive rights to provide an alternative entry point to understanding how the embodied experience of the sterilization unfolded in women's lives.

## Funding

Wellcome Collaborative Award number 209829/Z/17/Z, Wenner‐Gren Foundation, Dissertation Fieldwork Grant 2015. Social Science Research Council, Dissertation Proposal Development Fellowship 2012.

## Notes

1 Ligadura, abbreviated version of ligadura de trompas (tubal ligation), is the term used in rural communities to refer to the operation.

2 Chacra, a loanword from Quechua, is a small plot of land for agricultural activities.

3 These sequelae resemble Christen Smith's (2016) sequelae of anti‐Black police violence in Brazil that she describes as the “lingering effects of violence” (Smith 2016: 149) that radiate beyond the victim into their families and communities.

4 The ombudsman, created in 1993, receives complaints, inquiries, and requests from citizens who have experienced a violation of their rights. Its role is not to judge or prosecute, but to document cases and provide recommendations to the corresponding authorities.
